# Subjective semantic surprise resulting from divided attention biases evaluations of an idea’s creativity

**DOI:** 10.1038/s41598-020-59096-y

**Published:** 2020-02-07

**Authors:** Goran Calic, Nour El Shamy, Isaac Kinley, Scott Watter, Khaled Hassanein

**Affiliations:** 1 DeGroote School of Business McMaster University 1280 Main Street West, Hamilton, Ontario L8S 4L8 Canada; 2 Department of Psychology, Neuroscience & Behaviour McMaster University, 1280 Main Street West, Hamilton, Ontario L8S 4L8 Canada

**Keywords:** Neuroscience, Cognitive neuroscience, Attention, Problem solving

## Abstract

The evaluation of an idea’s creativity constitutes an important step in successfully responding to an unexpected problem with a new solution. Yet, distractions compete for cognitive resources with the evaluation process and may change how individuals evaluate ideas. In this paper, we investigate whether attentional demands from these distractions bias creativity evaluations. This question is examined using 1,065 creativity evaluations of 15 alternative uses of everyday objects by 71 study participants. Participants in the distraction group (Treatment) rated the alternative uses as more creative on the novelty dimension, but not the usefulness dimension, than did participants in the baseline group (Control). Psychophysiological measurements—event-related and spectral EEG and pupillometry—confirm attentional resources in the Treatment group are being diverted to a distractor task and that the Control group expended significantly more cognitive resources on the evaluation of the alternative uses. These data show direct physiological evidence that distractor tasks draw cognitive resources from creative evaluation and that such distractions will bias judgements of creativity.

## Introduction

When the air force commissioned J. P. Guilford, a psychologist at the University of Southern California, to conduct one of the first studies of creativity^1^, it was to help select pilots who in an emergency situation – the unexpected failure of an instrument or gear – would respond with an appropriately creative solution and save themselves and the airplane. Creative ideas continue to be especially valuable in unexpected situations that have already pushed information processing to its limiting point^2^. Today, researchers describe the challenges of an interconnected and technology mediated workplace on cognitive performance. On average, a business person sends and receives 109 e-mails per day and that rate is growing at 7% per year^3^. Instant messages sent and received are similarly increasing, at 11% per year^3^. Even short distractions can be detrimental to cognitive performance. A distraction lasting 2.8 seconds, about the length of a smartphone push notification, can double the risk of decision error^4^. As such, it may be as important to study factors that bias creativity as it is to study creativity^5^. An understanding of factors that bias creativity can lead to remediation of such factors, therefore contributing to improvements in peoples’ ability to solve problems^6^.

Evaluation of ideas constitutes an important stage of the creativity process^7–9^. Yet, evaluating the creative potential of ideas is not a trivial stage of the creative process as it requires attention, which is taxed by task-unrelated stimuli^10–12^. Distractions, defined as task-unrelated cognitive load, that compete for cognitive resources by drawing attention away from evaluations may result in different evaluations than the individual would make when free from distracting stimuli, thus resulting in suboptimal selection of problem solutions.

We hypothesize that distractions that draw attention away from the evaluation of ideas will result in changes in creativity evaluations. These evaluations differ from evaluations about an idea’s creativity that individuals make in a state free of distractions. Thus, *this study sets out to test whether distractions bias creativity evaluations*. *More specifically*, *we present the hypothesis that attentional demands from distracting stimuli will result in upward biased creativity evaluations*, *such that ideas are evaluated as more creative than they would be by individuals free from distraction*.

We rely on behavioral methods, that is creativity evaluations of alternative uses (AUs), to test the broad hypothesis that distractions bias creativity evaluations. We leverage psychophysiological methods such as pupillometry and event-related (ERP) and spectral electroencephalography (EEG) to verify that distracted individuals are expending less cognitive resources on the evaluation task. The three methods are complimentary. Established pupillometry, EEG oscillations, and ERP components (e.g., alpha band desynchronization, P300) provide reliable and robust real-time measures of attention and cognitive load^13,14^ that is not susceptible to retrospective and subjectivity biases^15^. Multi-method experiments provide support for the existence of a bias.

## Biases In Creativity

Selecting among alternative ideas is a fundamental challenge for individuals in a number of different organizational settings^16,17^. To this end, people devote a great deal of time and effort to evaluating ideas. Sevens and Burley^18^ find that, on average, managers evaluate more than 3,000 raw ideas to identify one that is commercially successful. The challenge of idea evaluation has only grown with the increase of ideation maximization training, platform-based contests, big data, and crowdfunding as a means of generating a large number of ideas^19–21^. The challenge faced by decision-makers is often to select the most creative idea from a myriad of competing alternatives and to reduce biases that may have a detrimental effect on this process^22^.

In social sciences, mathematics, and engineering, biases refer to systematic errors impacting performance^23^. Prior research has identified a number of decision-making errors in relatively simple decision-making tasks^24,25^. Research has also examined biases present in creativity^6,26^. Broadly, this research has examined biases against or about creative ideas. For instance, Mueller, Melwani, and Goncalo^27^ explain that people often reject creative ideas, despite espousing creativity as a desired goal, in an effort to reduce uncertainty. As another example, Glăveanu^28^ finds partial support for an “art bias”, which is the implicit association between art and creativity. Glăveanu finds that artistic professions are scored highest in terms of creativity as a key requirement. Researchers have also discovered biases during stages of the creative process. In examining idea evaluation, Blair and Mumford^29^ find that people disregard original, risky, and time-consuming ideas, even when instructed to select ideas for new programs. In another study, Licuanan, Dailey, and Mumford^30^ find that when active analysis of an idea’s originality was encouraged, people selected more original ideas. These studies highlight that priming individuals can influence both how they think about creativity and how they perform during a creativity task. Using this research as a starting point, we build on work about factors affecting idea evaluation by exploring whether surprise, which is the presentation of an idea that was unexpected by the individual, impacts creativity evaluations^31,32^.

### Surprise bias

Creative ideas are synthesized by reconfiguring knowledge in new ways (i.e., the novelty parameter) to achieve a particular objective (i.e., the usefulness parameter)^7,33–35^. Recently, Simonton^31^ also used surprise to conceptualize creativity. In Simonton’s^31^ recent and Campbell’s original^36^ conceptualization of blind variation selective retention (BVSR) theory, creativity and its sub-component of “surprise” are subjective rather than objective parameters. In fact, Campbell’s original definition of BVSR focused on “thought trails” occurring within a person’s head^37^. It follows from these conceptualizations that if creativity is subjectively experienced then differences in experience at the presentation of an idea can result in differences in the evaluation of the creativity of the same idea.

Simonton’s^31^ three criterion definition (novel, useful, and surprising) allows the parameters to vary independently to acknowledge their separate impact on creativity. This is a useful feature, particularly because it allows creativity researchers to define ideas with more precision and comprehensiveness^32^. That the parameters of novelty, usefulness, and surprise are allowed to vary independently does not preclude their interdependence—a change in one parameter resulting in the change of another. This is intuitive when one considers the subjective nature of creativity just discussed. Instead of conceptualizing creativity as jointly determined by novelty, usefulness, and surprise, we can conceptualize the creativity function (creativity equals the product of novelty, usefulness, and surprise) as endogenous, with experiences of creativity also determining the levels of novelty, usefulness, and surprise experienced by an individual. Assuming endogeneity, by manipulating any of creativity’s subcomponents, one can also manipulate the other two components. Mathematically, this is the equivalent of restructuring Simonton’s creativity equation to solve for novelty, usefulness, or surprise, rather than for creativity^31^.

The specific example used by Simonton^31^ to demonstrate the subjective and endogenous characteristics of creativity is the invention of the Pelton water wheel, conceived by simultaneously two independent inventors^38^. Although the inventions were objectively identical, equally novel and useful, one inventor adapted the idea from a previous invention and the other inventor unknowingly came up with the same idea after a lucky event, rendering the idea for the invention highly surprising to him. Only the latter sought patent protection and got credit for the invention, possibly because, by the current definition, the latter experienced a subjectively higher degree of creativity resulting from a higher feeling of surprise, and thus also novelty and usefulness, and as such chose to act on his hunch that he was onto something.

Thus, our research interest is situations that influence the evaluation of creativity by eliciting surprise at the presentation of an idea. Note that we do not intend “surprise” here to imply a relationship or equivalence with low-level attentional alerting or orienting responses, such as when a sudden loud noise involuntarily attracts attentional focus. Rather, we use “surprise” here to describe how the lack of deliberate controlled evaluation (or in more colloquial terms, a lack of sufficient “thinking about”) an event or object can make related information relatively unexpected. Due to their ubiquity and increasing prevalence, we focus on distractions to bias creativity evaluations in this way. Distractors will focus information processing on the (distracting) task at hand, limit information processing for creativity evaluation, and result in higher surprise to presented ideas. We posit that this subjective degree of semantic or conceptual surprise at these related ideas will be accompanied by a higher subjective experience of creativity^31^.

Thus, we formulate the surprise bias hypothesis:

Task-unrelated cognitive load—distractions—will result in higher creativity evaluations.

## Methods

### Sample and procedure

Analyses for this paper were based on a sample of 1,065 idea evaluations from 71 participants (26 female, 45 male). The participants were recruited from McMaster University’s staff and student pools, and varied in both age (*M* = 28, range 21–59) and educational backgrounds (High School, = 5, College = 4, Bachelors = 43, Master’s = 17, PhD = 2). Approval for the study was obtained from the McMaster Research Ethics Board (MREB) and the study was carried out in accordance with the relevant guidelines and regulations. Informed consent was obtained from all participants prior to the study. Each participant evaluated the creativity of the same 15 ideas, which were alternative uses, drawn from previously published creativity research, for each of three everyday objects (tin can, brick, and ping pong ball)^39,40^. Alternative uses were evaluated on a scale of 1–5 (1 = strongly disagree, 5 = strongly agree) on the dimensions of novelty and usefulness, which were summed for the creativity variable. Participants were paid for their time and the experiment lasted 20 minutes on average.

Participants were randomly assigned to either a baseline (n = 39, 15 female) or a distraction (n = 32, 11 female) group. In the distraction group (i.e., Treatment), participants simultaneously performed an auditory oddball task and evaluated the 15 AUs of everyday objects. In the present auditory oddball task, auditory stimuli, alternating between a rare high-pitched tone (n = 17) and a frequent low-pitched tone (n = 133), were played every second (tone duration = 100 ms, rise and fall duration = 30 ms, total duration = 160 ms) for both groups during the AU tasks. The sounds used were dual-tone multi-frequency (DTMF) signaling tones that are standard in telecommunications, and the tones denoting the numbers 1 and 9 were used for the low- and high-pitched tones, respectively.

Participants in the Control group were asked to ignore the target tones, while those in the Treatment group were asked to silently count the number of times the target (high-pitched) tone was played and to disregard the non-target tones. At the beginning of the experiment, participants in the Treatment group were given the opportunity to practice and identify both the target and noise tones as many times as they wanted by pressing one of two keyboard keys assigned to play each tone. Once the participants in the Treatment group were done practicing and indicated that they were ready to start the experiment by pressing a third key, a message instructed them to get ready for the actual experimental tasks by clearing their memory and starting a fresh count of the target tones (i.e., Clear your memory. Start a new Target count from 0 for the upcoming task. Press a key to begin the task)^41^. Participants then engaged with a set of five AU evaluation trials for the first of three objects. For each trial, one AU for the object was displayed for 10 seconds, while the tones were simultaneously played at 1 second intervals starting at the onset of the AU. All participants were then asked to rate the novelty and usefulness of the AU of that object.

**Figure 1:**
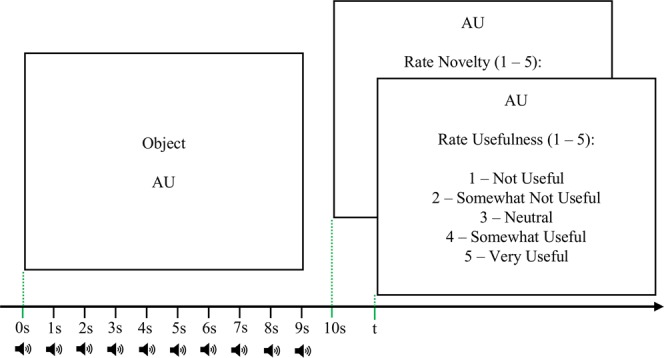
Experimental Paradigm. For each of the 5 AUs for each of the 3 objects (i.e., total of 15 trials), the Object and AU were displayed for 10 seconds. Simultaneously, the auditory oddball stimuli were played every 1,000 milliseconds (n = 10) starting at the onset of the AU, randomized between rare target (i.e., high-pitched) and frequent non-target (i.e., low-pitched) tones.

After completing five AU evaluation tasks (i.e., trials) for one everyday object, participants in the Treatment group indicated the number of times the high-pitched tone (i.e., target) was played. Participants in the Treatment group were then again asked to clear their memory (i.e., reset counting target tones in their mind and restart from 0) and to get ready for a new set of AU’s (i.e., trials) for a new everyday object. Figure 1 provides a summary of the trials. After completing the experiment, all participants were asked to indicate how distracted they were by the auditory stimuli on a scale of 1–5 (1 = not distracting at all, 5 = very distracting).

Given the importance of cognitive work to our study, we utilize a non-intrusive behavioral manipulation check of attention. Manipulation checks are particularly important when participants are not responding to an involving situation, but rather sitting at a computer pressing keys in response to written stimuli^42^. Previous studies find that over 30% of participants in non-involving situations failed an Instructional Manipulation Check (IMC)^43^, also called an attention check, a screener, or a trap question. They also found that only those participants that passed the IMC were affected by the treatment. Because explicit manipulation checks can amplify, undo, or interact with the effects of a treatment, recent research suggests non-intrusive behavioral checks of attention^44^. We follow this method in the current study. Participants that pressed the same keyboard key combination for one third or more of all novelty and usefulness ratings were excluded from the analysis. Ninety-two participants were recruited for the study and 21 (23%) failed the manipulation check.

### Psychophysiological measures

We used ERP analysis, spectral EEG analysis, and eye tracking methods to test the hypothesis that biased creativity evaluations are associated with divided attention from distracting stimulus Treatment. During the AU experiment, continuous EEG signals were recorded from 20 channels arranged according to the international 10–20 system using Cognionics Quick-20 Dry EEG headsets at 500 Hz. Eye tracking data, including fixations and pupil diameter, were collected using the Tobii Pro X2–60 at 60 Hz. Participants were screened for eye health related problems such as glaucoma, cataracts, the need for bifocals or other assistive technologies.

### EEG preprocessing

Continuous data were first bandpass filtered between 1 and 30 Hz using a Hamming windowed sinc FIR filter as implemented in EEGLAB (version 14)^45^. Noisy channels were removed on the basis of low correlation (below 0.8) with adjacent channels. Artifact subspace reconstruction with a cutoff parameter of 5 was then used to clean the continuous data^45^. Missing channels were then interpolated using EEGLAB’s spherical dipole fit function^46–48^. Data from 8 participants was discarded due to technical issues during recording.

### Event-related potential analysis

For ERP analysis, epochs were extracted from 200 ms prior to the onset of auditory stimuli to 700 ms after. Baseline correction was performed using pre-stimulus period. Epochs containing artifacts were marked using the moving window peak-to-peak threshold function as implemented in ERPLAB, with a threshold of 100 uV, a window width of 200 ms, and a step size of 100 ms^13^. ERPLAB’s function for detecting step-like artifacts resulting from saccades was also applied with a threshold of 100 uV, a window width of 200 ms, and a step size of 50 ms^49^. Epochs in which any channel triggered either artifact detection function were excluded from further analysis. In total, there were 4916 valid epochs (4358 non-target audio, 558 target audio) in the Control group and 4403 (3902 non-target audio, 501 target audio) in the Treatment group.

### Spectral EEG analysis

For spectral analysis, 10-second epochs were extracted for the duration of object display. The same artifact rejection procedure as in the ERP analysis was applied to these epochs. For each epoch, power spectral density was estimated at frequencies separated by steps of 0.1 Hz using rectangular-windowed periodograms. The spectral densities were log-transformed and averages between frequency limits of these log-transformed values were used for subsequent statistical analysis.

### Pupillometry

Pupil dilation is widely established as an indicator of cognitive state during task performance under equiluminant conditions^50–52^. Specifically, pupils involuntarily dilate as a sympathetic response of the autonomic nervous system (ANS) proportional to the degree of mental workload or attentional/effortful engagement with the cognitive demands of a task^24,50,53^.

Pupil diameter data for the left and right eyes were collected over the AU experiment, sampled continuously at 60 Hz. Missing pupil data, typically due to blinking, were linearly interpolated after removing two samples before and after the missing data segment, to avoid measurement artifacts from half-occluded pupils during blinking. Data were initially smoothed by a 7-point moving average filter, then left and right pupil data were averaged to provide a single pupil dilation dataset. Two analysis datasets were defined: (i) 9000 ms trial segments from the AU stimulus presentation onset, baselined to the average pupil diameter in the preceding 2000 ms epoch; and (ii) 2000 ms segments based on auditory stimulus onsets for sounds 2 to 9, excluding the AU onset sound and the final sound, within AU trials, with a 300 ms pre-stimulus baseline. Baselining subtracted the mean baseline pupil diameter value from the trial epoch data, and then divided the epoch data by this baseline value; this provides pupil dilation data as a proportion of change from baseline. In addition to baseline-adjusted data, we also assessed the same data as absolute pupil diameter measures (no baseline adjustment) to capture differences between conditions that could persist beyond single AU judgements, particularly the continuous cost of dual-task monitoring in the Treatment condition compared to single-task Control. Trials with more than 50% missing/interpolated values in the original unprocessed data were excluded from analysis, suggestive of sub-optimal data recording beyond participant blinks. Slight differences in the number of included AU-onset trials with different analysis durations is the result of applying the above exclusion criteria on whole-trial data segments with varying lengths.

### Statistical analysis

For behavioral, EEG, and pupil dilation data, we used linear mixed effects models with restricted maximum likelihood (REML) estimation to better control for random effects of participants and/or items, as available data and study design allowed. We used the “mixed” function from the “afex” package^54^, in R software^55^, which is a recent adaptation of the commonly-used “lme4” package^56^. We describe the particular fixed and random factor structures of these models for particular datasets, below. It is important to note that estimated denominator degrees of freedom for *F* or *t* statistics from these models are not readily interpretable as is typically expected from typical ANOVA models; instead we report the number of individual observations for dependent measures, along with numbers of participants, items, and factor levels for each analysis. We report estimated *t* statistics and associated *p*-values calculated using Satterthwaite’s method, recently shown to be a reliable estimator of Type 1 error rates across both small and large sample sizes^57^. All tests for these and other analyses were two-tailed.

## Results

### Behavioral measures

Participants in the Treatment group rated the auditory stimuli as more distracting than the participants in the Control group, *t* = 2.77, *p* = 0.007, verifying our distraction manipulation. Creativity ratings were developed for each AU by summing the novelty and usefulness ratings together^58^. The final sample results in 1,065 idea evaluations, 480 for the Treatment (n = 32) and 585 for the Control (n = 39) group. We assed these data using a mixed linear model with a fixed effect of group (Treatment versus Control). Considering rating differences between groups of participants with our distraction manipulation was our primary behavioral measure, we controlled for the random effect of item (n = 15; intercepts only, not slope), considering the wide variability of ratings across different individual AU items. Participants in the Treatment group rated the creativity of AUs as higher than the participants in the Control group, supporting the existence of upward biased creativity ratings *t* = 2.41, *p* = 0.016. We also explored the difference in AU evaluations for the two dimensions of creativity. Both ratings for usefulness and novelty were higher in the Treatment group, but only the novelty ratings were statistically significant for the Treatment group, *t* = 2.37, *p* = 0.018, while those for usefulness were not *t* = 0.86, *p* = 0.389.

The significant difference in creativity evaluations between the distraction and base-line group provides initial support for the existence of a surprise bias. However, the differences presented in Table 1 do not address the question of whether these effects are because of divided attention, accompanied by surprise. To make progress in this direction, we measured differences in attentional demand between the two groups.

**Table 1:** Means and Standard Deviations for Creativity Assessments.

	Distraction manipulation check	Ratings of Alternative Uses		
		Novelty	Usefulness	Creativity
Treatment (T)	3.03	3.10	3.19	6.30
n = 32	(0.98)	(1.47)	(1.53)	(1.66)
Baseline (B)	2.36	2.92	3.13	6.05
n = 39	(1.03)	(1.48)	(1.51)	(1.72)
T ≠ B	*t* = 2.77	*t* = 2.37	*t* = 0.86	*t* = 2.41
	*p* = 0.007	*p* = 0.018	*p* = 0.389	*p* = 0.016

### Event-related EEG

**Figure 2:**
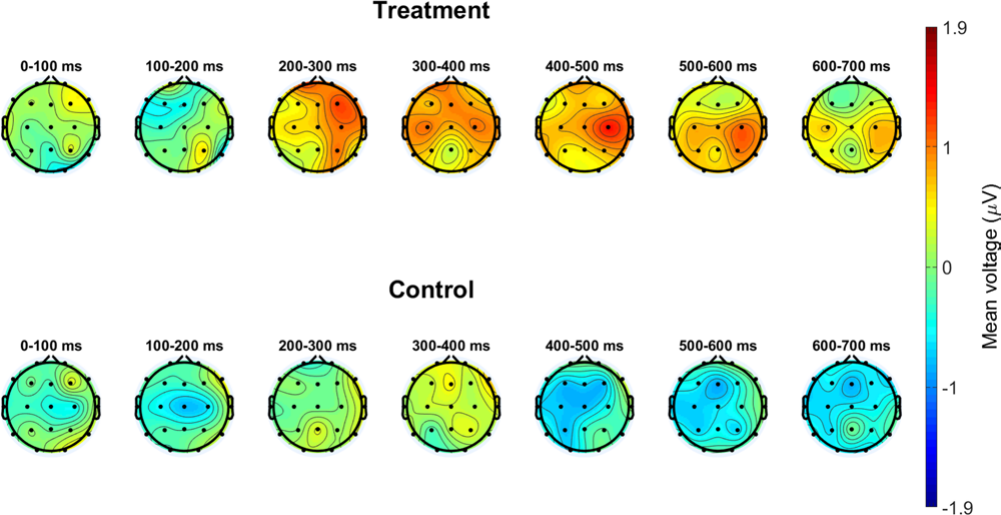
Scalp topography of response to target audio. Data points are the average voltage at the given electrode over the given 100-ms post-stimulus period. The group performing the counting task showed a late, positive, P300 response, indicating that they were attending to the audio stimuli.

**Figure 3:**
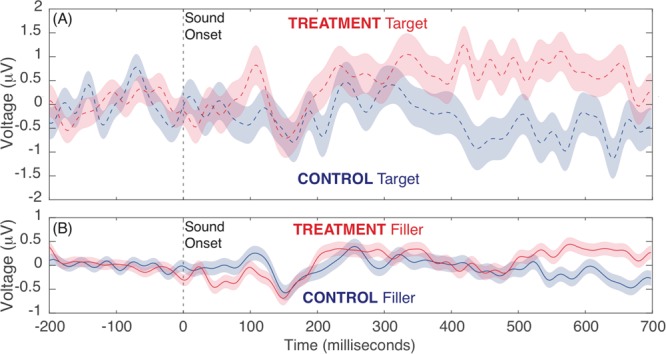
ERP waveforms in response to Target (Panel A) and Filler (non-target, Panel B) audio tones. Waveforms are grand averages over central and parietal electrodes (Cz, C3, C4, Pz, P3, P4, P7, and P8). Shaded error bars represent one SEM.

The Treatment group showed a late, positive centroparietal response to the target audio with polarity, latency, and topography consistent with the P3b component. The Control group showed no such response to the target audio. These results are presented in Fig. 2, which shows each group’s scalp topographies in response to the target audio, and Fig. 3A, which shows each group’s ERP waveform in response to the target audio. For statistical comparisons, average voltages from 400 to 600 ms post-auditory stimulus onset at central and parietal electrodes (Cz, C3, C4, Pz, P3, P4, P7, and P8) were computed. The Treatment group’s response to the target audio was greater than the Control group’s response to either the target or the non-target audio, or the Treatment group’s own response to the non-target audio, which is presented in Fig. 3, Panel B. We assessed these data using a linear mixed effects model with fixed effects of group (Treatment versus Control) and audio type (Target versus Filler), controlling for the random effect of participant (intercepts and slopes for both group and audio type effects). Analyses included 9319 total observations (mean P3b amplitude on a single trial) from 66 participants (five participants had no usable EEG trials for P3b analysis). The enhanced attentional P3b effect for Target audio for the Treatment group was supported by the significant interaction between group and audio type, *t* = 2.61, *p* = 0.009, in addition to driving an overall main effect of larger P3b responses for the Treatment group, *t* = 3.25, *p* = 0.002. There was no main effect of audio type, *t* = 0.51, *p* = 0.609.

According to the context updating theory, which suggests that the brain constantly and automatically generates hypotheses about the environment and imminent experiences, the P300 component results from the attention-driven process of comparing the previous event in working memory to the current one^14,59^. If a change is detected, the stimulus context is updated and a P300 component occurs. Indeed, the P300 has been used as an indicator of whether stimuli have broken through an “attentional barrier”^60^. Thus, the presence of a P300 response to target audio stimuli in the Treatment group is evidence that the context-updating process induced by the audio counting task was drawing attentional resources away from the creativity evaluations.

### Spectral EEG

There are numerous reports that parietal alpha power decreases and frontal midline theta power increases with increasing task load^10,61–63^. Alpha desynchronization (i.e., decrease in alpha power) is thought to reflect the process of information retrieval, whereas theta oscillations are thought to result from the activation of hippocampo-cortical feedback loops and reflect the encoding of new information as well as general task demands^63,64^.

**Figure 4:**
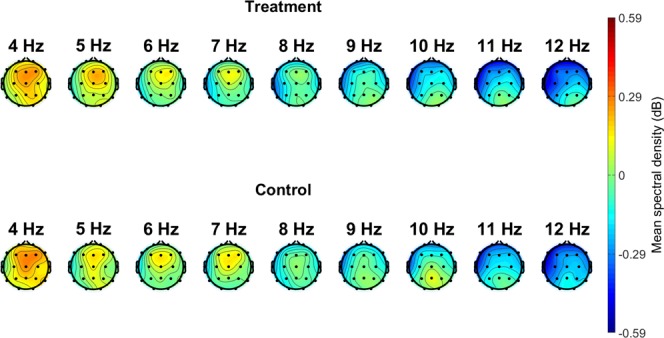
Topography of spectral densities at various frequencies. Data points are the average periodogram values at the given frequencies and electrodes, as calculated over the 10-second epochs during which objects were presented. Both groups show a frontal theta concentration and a parietal alpha concentration.

Figure 4 presents average periodogram values at given frequencies and electrodes, as calculated over the 1 second epoch after the target audio stimuli began. Both groups showed a frontal concentration of theta power (4–7 Hz) and a parietal concentration of alpha power (8–12 Hz). We assessed these data using a linear mixed effects model with a fixed effect of condition (Treatment versus Control), controlling for the random effect of participant. In total, 756 trials from 62 participants were included in the analyses. Participants were excluded from this analysis if their Fz or Pz channels had been interpolated in preprocessing. Compared to the Control group, the Treatment group had significantly lower parietal midline alpha (10 Hz) power, *t* = 2.60, *p* = 0.012, although there was no difference in frontal midline theta power (4–7 Hz) between the groups (*t* = 0.18, *p* = 0.857). Taken together, the ERP and spectral analysis results serve as a manipulation check and demonstrate that the auditory counting task was drawing attentional resources in the Treatment group.

### Pupillometry

**Figure 5:**
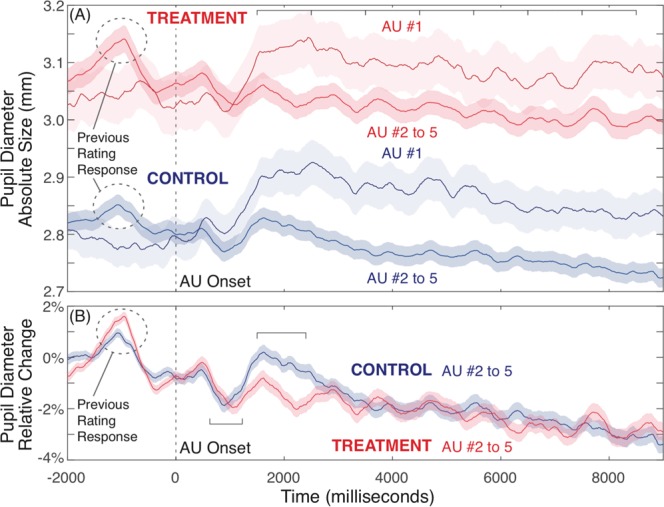
Mean pupil dilation data for the AU analysis task. Panel A shows absolute pupil diameter over time, with larger pupil dilation for dual-task Treatment versus single-task Control, suggesting overall greater cognitive work with continuous auditory monitoring in addition to AU evaluation. Panel B shows relative change of pupil dilation over time, relative to pre-stimulus baseline mean, for AU #2 to 5 data (excluding the initial AU for an item). The event-related AU pupil response (beginning ~1500 ms) was relatively larger for Control versus Treatment. Control participants were able to invest greater cognitive work for deliberate focused event-related AU evaluation relative to background non-AU task demands. While Treatment pupil responses were larger overall with ongoing dual-task demands (Panel A), event-related pupil dilation (Panel B) was smaller for AU-specific cognitive work for Treatment participants, suggesting a direct measure of reduced cognitive evaluation of AUs under distraction, predicting biased evaluations. Brackets indicate data analysis time windows. Shaded error bars indicate one SEM.

Figure 5 shows mean pupil dilation data over time for Treatment and Control groups. Panel A shows absolute pupil size (diameter, mm) separately for the first AU trial (AU #1, immediately following the item definition) in each five-AU set for a given item, and then for the mean of the subsequent four AUs for that item (AU #2 to 5, where each immediately follows the rating responses for the previous AU definition). Panel B shows this latter mean AU #2 to 5 pupil dilation data as the relative change in diameter from baseline, to better visualize the relative event-related changes on top of background pupil dilation effects for Treatment versus Control conditions.

We assessed absolute pupil size data with a mixed linear models approach, with three fixed factors: group (Treatment versus Control), to capture an overall measure of dual-task load and distraction; Trial Type (AU #1 versus AU #2 to 5), to observe any differences in initial item evaluation from later more isolated measures of AU evaluation; and Time Window (mean data within seven contiguous 1000 ms windows starting at 1500–2500 ms, up to 7500–8500 ms), to assess the timecourse of AU-related pupil dilation effects. The model included participants as a random factor. A full model including random slopes for the effects of both group and trial type factors would not resolve; we computed models with one or the other terms independently, which showed extremely similar results, and we report the most conservative results here. These analyses included 5964 total observations, across the combination two conditions by seven time windows, and available trials of pupil data from 64 participants.

From Fig. 5A, the Treatment group showed an overall greater pupil dilation effect, *t* = 2.46, *p* = 0.017, consistent with a generalized greater cognitive load for the continuous dual-task monitoring requirements of the Treatment condition, compared to Controls who had no ongoing sound monitoring requirement. On top of this general group effect, both groups showed a strong additional pupil dilation effect for the first AU item compared to subsequent AU evaluations, *t* = 10.04, *p* < 0.001. This initial larger response likely represents additional evaluation and semantic representation of the item itself (as well as the AU evaluation), once there is an initial specific cue to drive that need for evaluation. Subsequent AU evaluations (AUs #2 to 5) likely represent a more focused and “pure” measure of the AU evaluation process itself. We also note the visible set of pupil dilation peaks at one second intervals in the Treatment AU #2 to 5 data (mostly absent in the equivalent Control data), driven by sounds in the auditory task when participants are monitoring for rare tones. Finally, the assessment of Time Window (estimated ANOVA-equivalent main effect considering all seven levels of this factor) showed AU-related pupil effects over time, *F* = 15.41, *p* < 0.001, with an initial maximal peak around 1500 to 2500 ms, that gradually diminished over the course of the trial. The linear contrast for Time Window from the equivalent 3-way Repeated Measures ANOVA showed a strong linear decreasing trend over time, *F*(1,61) = 24.71, *p* < 0.001. These main effects of group, Trial Type and Time Window did not show any significant evidence of interactions, all *F*s < 1.25.

To better assess AU-specific processing differences between Treatment and Control groups, we analyzed differences over particular time windows for mean AU #2 to 5 data, to more carefully quantify the event-related pupil dilation differences representing the immediate focused cognitive work of AU evaluation. Figure 5B shows relative changes in pupil data for AU #2 to 5 trials, baselined to the 2000 ms period before the AU onset. Following the AU onset, there is an initial positive dilation peak around 600 ms (a response to the initial tone that accompanies the AU onset), then a relaxation/constriction around 1000 ms, followed by the onset of a sustained dilation response beginning around 1500 ms that corresponds to the cognitive work of AU evaluation. The delayed timecourse of 1 to 1.5 seconds is typical of event-related pupil responses to complex evaluative tasks.

Given the relative amplitude offset between the Treatment and Control groups could be influenced by differences in pre-stimulus baseline, we assessed peak-to-peak minimum-to-maximum mean amplitude differences to compare groups. We assessed mean absolute pupil diameter data over a 1500 to 2400 ms time window capturing the maximal AU-related dilation effect, subtracting the mean of the preceding relaxation/constriction minimum over a 700 to 1300 ms window (analysis epochs shown in Fig. 5B). A mixed linear model with a fixed effect of group, controlling for the random effect of participant (independent intercepts only, no stable model identifiable with group by participant interaction), used 672 observations from available pupil data from 64 participants. The Control group had a significantly larger AU-related pupil dilation response versus Treatment, *t* = 2.45, *p* = 0.018. While we observe substantially greater sustained pupil dilation with more ongoing cognitive work and dual-task monitoring in the Treatment condition (Fig. 5A), the additional cognitive work on top of this ongoing background effort is larger for Control than Treatment participants. These data suggest that participants in the Treatment condition expend considerably more cognitive effort throughout the task, and that their ability to devote mental resources to AU evaluation (here as indexed by pupil dilation specifically time-locked to AU evaluation processes) is observably diminished compared to Control participants with lower ongoing cognitive demands. We also note that the pattern of pupil dilation over time for Treatment participants is strongly locked to the timing of tone stimuli, suggesting that the specific AU evaluation response may be even less than we measure here. These data give direct physiological evidence that Treatment participants are devoting relatively less deliberate cognitive work to evaluating AU items.

**Figure 6:**
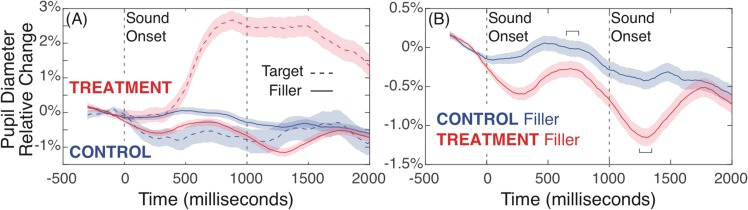
Pupil responses for sound stimuli onset, for sounds 2 to 9 during the AU trial (one per second, beginning at 1000 ms). Panel A shows the expected large pupil dilation response for attended Target sounds for the Tretament condition. Panel B shows pupil dilation responses to auditory Filler (non-target) stimuli. The Treatment condition (counting Target tones) showed a larger response to non-target sounds versus Control (ignoring tones), confirming a greater continuous overall attentional investment in the secondary task in the Treatment group. Brackets indicate data analysis timewindows. Shaded error bars indicate one SEM.

In addition to pupil dilation effects relative to AU evaluation, we also assessed pupil dilation responses to auditory tone stimuli directly, as an additional converging measure of the relative attentional load and distraction involved with our Treatment condition. Figure 6 shows relative pupil dilation responses to Target (rare, high pitched) and Filler (common, low pitched) sounds in Panel A. The enormous difference in pupil response to Target sounds between Treatment and Control conditions is expected, given Treatment participants were explicitly monitoring for and keeping count of these rare events. A more useful comparison is shown in Panel B, considering only pupil responses to Filler sounds – this reveals a more subtle but very important difference between Treatment and Control task demands. For Filler sounds we observe a strong entrainment of pupil dilation effects in the Treatment condition versus Control, even though Treatment participants don’t need to do anything with these Filler stimuli. The attentional and task demands to simply monitor the regular auditory tones for rare targets is enough for a substantial attentional effect to be seen for Treatment participants for non-target stimuli under these conditions.

As before, given the relative amplitude offset between the treatment and Control groups could be influenced by differences in pre-stimulus baseline, we again assessed peak-to-peak minimum-to-maximum amplitude differences to compare groups. We assessed the audio tone Filler pupil effect as the difference between mean maximum and mean minimum absolute pupil diameter values in 650–750 ms and 1250–1350 ms time windows, respectively, best capturing the peak dilation and constriction periods in these data. A mixed linear model with a fixed effect of group, controlling for the random effect of participant (independent intercepts only, no stable model identifiable with independent slopes), used 4607 observations from available pupil data from 65 participants. Treatment participants had a significantly larger Filler tone-related pupil response, *t* = 2.63, *p* = 0.011. The size of this pupil dilation response is approximately a 1% change in diameter, which is quite substantial considering the AU-related pupil response for Control participants is not much more than a 2% change (though sustained over a longer period of time).

## General Discussion

This study confirms the surprise bias hypothesis. The surprise bias hypothesis states that distractions bias an individual to judge an idea as more creative than an individual would in a state free of distraction. Participants in the Treatment group rated the AUs as more creative on the novelty dimension, but not the usefulness dimension, than did participants in the Control group. Psychophysiological analysis confirmed the Treatment group was dividing attentional resources during the experiment and that the Control group expended more cognitive resources on evaluation of AUs.

### Neural basis of creativity evaluation

We contribute to the literature on creativity with neural basis for creativity evaluation. Our findings suggest that cognitive load biases an individual’s rating of novelty but not their rating of usefulness. This suggests differences in cognitive work required to evaluate novelty and usefulness of ideas. Evaluations of usefulness are contingent on the individual’s appraisal of whether an idea satisfies an applicable condition of subjective meaningfulness, such as utility or aesthetic appeal. As such, evaluations of usefulness are based on a tests of whether the idea solves a particular problem and the outcomes of such tests are often dichotomous (the idea is useful, or it is not)^31^. In contrast, evaluations of novelty require more cognitive work, possibly because novelty evaluations are judged against the probability the evaluator could generated the presented idea^31^. The less likely the evaluator is to generate the presented idea, the more likely they are to rate that idea as novel. Thus, the statistically significant biasing effect of distractions on novelty ratings, but not on usefulness ratings, indicates that accuracy of novelty evaluation is more susceptive to distractions.

The implications is that usefulness evaluations are less cognitively demanding than are novelty ratings. For instance, the usefulness of the Pythagorean theorem to solve the relation among the three sides of a triangle can be judged based on the empirical test: Does the triangle have a right angle? The theorem is useful for all right triangles and useless for all other triangles. As such, the evaluation of whether the Pythagorean theorem is useful is as simple as identifying a right triangle. Similarly, the usefulness to Duncker’s creativity candle problem^65^ can be judged based on the empirical test: Is the candle affixed to the wall and does it drip wax on the table? A solution either affixes the candle to the wall so that it does not drip wax on the table, or it does not. Thus, our findings indicate that drawing attentional resources from a creativity evaluation task is particularly likely to bias assessments of novelty.

Distractions bias creativity evaluations in a specific direction: upward. We suggest upward biased evaluations are the result of subjective experiences of surprise at the presented idea. According to blind variation and selective retention thoery^31,32,66^, experiences of creativity, which are associated with “Eureka!” and “Aha!” moments, are highly related to subjective feelings of surprise. It follows from this that conditions which elicit surprise at presented ideas should result in increased experiences of creativity. Within the context of the focal experiment, pupil dilation response is a marker of greater subjective semantic surprise or expectancy violation^67,68^. The substantially larger pupil dilation response in the Treatment group, compared with the response in the Control group, suggest higher experienced surprise at the presented AUs. Attentional elicitation and surprise are particularly closely related during creativity evaluation, where attentional demands of a distraction could draw cognitive resources away from evaluation, resulting in surprise at a presented idea, which leads to upward biased experiences of creativity. Taken together, our findings show that evaluations of novelty are cognitively more demanding, and thus more likely to be upward biased, than are evaluations of usefulness and that surprise (expectancy violation) is one possible source of this bias.

### Distracting stimuli bias creativity evaluations

Event-related and spectral EEG demonstrate that the counting task was drawing attentional resources in the Treatment group, but not from the Control group. The Treatment group showed a P3b component response to the target audio, which suggests attention-drive processes of comparison between two events in working memory (the repeating sound events). The Control group showed no such response. Additionally, linear mixed models of the ERP data show a significant interaction between condition and audio type. Compared to the Control group, the Treatment group had significantly lower parietal midline alpha power, suggesting increased task load.

Taken all together, pupil dilation results paint a coherent and converging picture of the cognitive demands and costs of ongoing dual-task monitoring in the Treatment, and the relative ability of participants in Treatment versus Control to selectively and adequately devote focused cognitive work to the AU evaluation task. Treatment participants showed substantially greater pupil dilation throughout the task, and substantially larger event-related pupil dilation to non-target auditory tone stimuli, both indicating a substantial, continuous, and effortful focus on the secondary tone monitoring task. Against this continuous background, Control participants showed larger event-related pupil responses specifically to immediate AU evaluations. These data converge to suggest that Control participants had more cognitive resources available to devote to deliberate focused AU evaluation, while Treatment participants were continuously working harder at a secondary task, and devoted fewer cognitive resources to AU evaluation even though their overall mental workload was higher throughout the task. These data corroborate the EEG findings suggesting the Treatment group was allocating attention to the distractor task. These data are also highly consistent with behavioral data, to show that reduced ability to focus on and sustain deliberative evaluation of AU items in the Treatment group leads to biased evaluation outcomes of creativity.

This study contributes to research exploring the impact of distractions on cognitive work. Distractions are detrimental to a wide variety of work that relies on focused attention. For instance, surgeons performing a laparoscopic cholecystectomy (i.e., gallbladder removal) were 44% more likely to make a surgical mistake when interrupted, and only 6% made mistakes when not interrupted^69^. Interruptive questions triggered the most errors, followed by background conversations. We extend the impact of distractions to evaluations of an idea’s creativity. Distractions bias creativity evaluations because they limit the cognitive resources available for processing information. We demonstrate that distractions bias the evaluation stage of the creative process in a specific direction and not only interrupt the generation of new ideas. Our work also suggests that optimizing cognitive resources, at least by removing distractions, will result in less biased creativity evaluations.

### Limitations and future research

The contribution of the current study should be considered in light of its limitation. One of the challenges in exploring the evaluation of ideas is the separation of the generation from the evaluation phase of the ideation process. In general, research assumes that ideas are, in fact, generated during the evaluation stage. For instance, in the Geneplore model of creativity, Finke, Ward, and Smith^70^ refer to evaluation as hypothesis testing, a stage during which the evaluator synthesizes and explores hypotheses (e.g., thought trails) about how the generated idea would interact with the environment in order to solve a problem. While we assume distractions interfere with this process in order to bias creativity evaluations, we do not directly test or observe these thought trials. Future research could explore how distractions interfere with hypothesis testing and to design remediations that facilitate idea evaluations.

While the use of low fidelity creative problem solving tasks, such as the remote associate tasks (RAT)^71^ and AUs^40,72–74^, is conducive to laboratory studies of creativity deploying methods sensitive to physical activity (e.g., EEG and pupillometry) and confounding factors (e.g. domain knowledge), more realistic measures of creativity in real-world problem solving would increase the external validity of the surprise bias^75^. Future research should look to replicate the current findings using more realistic measures of creativity, particularly those activities that reflect important components of creative ability, such as domain specificity and expertise. Distractions are detrimental to work performance and come in many guises that could affect creative performance. Proposals put forth by shareholder distract executives^76,77^ and background conversation distracts physicians performing surgery^69^. Future research could explore a variety of more realistic and context specific distractions as well as their effect on creative evaluations.

Perceived creativity influences idea selection and thus has practical implications, such as which ideas are ultimately allocated resources and implemented. As in the Pelton water wheel example, only the inventor who perceived the idea to be creative sought patent protection and received credit for the invention, even though both inventors had discovered objectively the same idea (i.e., identical novelty and usefulness). The study of whether and how biased evaluations impact idea selection offers opportunities for future research. An increase in the perceived creativity of ideas can lead to more ideas being considered for implementation, and eventually to the better idea being selected from the idea set. Alternatively, increases in perceived creativity can result in conventual ideas crowding out the more creative ideas, resulting in less creative idea being selected for implementation.

The implications of our findings are therefore unclear for practical purposes, particularly in light of recent research suggesting that intuitive, rather than deliberate, processing outperformers creative idea selection^78,79^. Given that dedication of attentional resources to task unrelated stimuli reduces an individual’s capacity for rational evaluation of an idea’s creativity^12^, distractions may simultaneously have biasing effects on idea evaluation and positive effects on idea selection. Consistent with these recent findings, researchers have suggested that relaxing evaluation criteria may result in more creative outcomes^9,80^. The possibility that distractions may, should they reduce deliberative processing and improve intuitive processing, enhance creative idea selection is an interesting question for researchers interested in which ideas are eventually acted upon. While the general underlying mechanisms between our and the just cited studies may be different, it is possible that, like in the Pelton water wheel example, higher levels of subjectively experienced creativity result in more idea selection. These are questions not explored in this study and opportunities for future research.

Lastly, the practical implication of the creativity bias is likely to be context specific. Organizations in film, music, writing, visual art, and other trademark, copyright, and patent-based industries value creative ideas to generate occasional, but highly profitable, blockbuster products^81,82^. In such organizations, extremes are what matter most^83^. In other settings, such as in manufacturing, incremental, but frequent, improvements to average product quality are what matters. Studying the effect of the surprise bias in different contexts will not only highlight the practical implications of biased creativity evaluations but address broader questions regarding the value of creativity evaluations.

## Conclusion

The results of this study demonstrate that distractions bias the evaluation of novelty. Distractions divide the evaluator’s attention and thus increase the likelihood the individual will evaluate an idea as more novel than they otherwise would. Because surprise is closely linked with experiences of creativity and because distractions reduce the possibility that an individual will generate a semantically similar idea, thus experiencing surprise, we refer to this phenomenon as surprise bias. These findings are confirmed by behavioral and psychophysiological analysis.

## Data Availability

The datasets generated during and/or analysed during the current study are available from the corresponding author on reasonable request.
